# Proton motive force underpins respiration-mediated potentiation of aminoglycoside lethality in pathogenic *Escherichia coli*

**DOI:** 10.1007/s00203-021-02710-y

**Published:** 2022-01-06

**Authors:** Calum M. Webster, Ayrianna M. Woody, Safura Fusseini, Louis G. Holmes, Gary K. Robinson, Mark Shepherd

**Affiliations:** School of Biosciences, RAPID Group, University of Kent, Canterbury, CT2 7NJ UK

**Keywords:** Aminoglycoside, *Escherichia coli*, Nitric oxide

## Abstract

It is well known that loss of aerobic respiration in Gram-negative bacteria can diminish the efficacy of a variety of bactericidal antibiotics, which has lead to subsequent demonstrations that the formation of reactive oxygen species (ROS) and the proton motive force (PMF) can both play a role in antibiotic toxicity. The susceptibility of Gram-negative bacteria to aminoglycoside antibiotics, particularly gentamicin, has previously been linked to both the production of ROS and the rate of antibiotic uptake that is mediated by the PMF, although the relative contributions of ROS and PMF to aminoglycoside toxicity has remained poorly understood. Herein, gentamicin was shown to elicit a very modest increase in ROS levels in an aerobically grown *Escherichia coli* clinical isolate. The well-characterised uncoupler 2,4-dinitrophenol (DNP) was used to disrupt the PMF, which resulted in a significant decrease in gentamicin lethality towards *E. coli*. DNP did not significantly alter respiratory oxygen consumption, supporting the hypothesis that this uncoupler does not increase ROS production via elevated respiratory oxidase activity. These observations support the hypothesis that maintenance of PMF rather than induction of ROS production underpins the mechanism for how the respiratory chain potentiates the toxicity of aminoglycosides. This was further supported by the demonstration that the uncoupler DNP elicits a dramatic decrease in gentamicin lethality under anaerobic conditions. Together, these data strongly suggest that maintenance of the PMF is the dominant mechanism for the respiratory chain in potentiating the toxic effects of aminoglycosides.

**Supplementary Information:**

The online version contains supplementary material available at 10.1007/s00203-021-02710-y.

## Introduction

It is well known that the abolition of aerobic respiration dramatically reduces the toxic effects of bactericidal antibiotics ((Lobritz et al. 2015); reviewed in (Stokes et al. 2019)). It is also accepted that the lethality of bactericidal antibiotics is linked to the activation of pathways that result in oxidative damage via elevation of aerobic respiratory rates (Dwyer et al. 2007, 2014; Foti et al. 2012), with superoxide causing destabilisation of iron–sulphur complexes (Imlay and Fridovich 1991; Keyer and Imlay 1996) leading to iron dysregulation and elevated Fenton chemistry. While different classes of bactericidal antibiotics have different primary cellular targets, most are able to induce the formation of hydroxyl radicals which is thought to be linked to the aerobic respiratory chain (Kohanski et al. 2007, 2010). The bactericidal aminoglycoside gentamicin directly targets the 30S subunit of the ribosome leading to protein mistranslation with the toxicity believed to be due to either irreversible uptake leading to complete ribosome inhibition (Davis 1987) or insertion of mis-translated proteins into the inner membrane (Davis et al. 1986). Evidence has suggested that mistranslated proteins brought to the membrane activate the two-component stress-response sensor Cpx which then may also activate ArcA a redox-responsive two-component transcription factor leading to respiratory and metabolic changes (Kohanski et al. 2008). These changes are proposed to lead to oxidative stress, production of hydroxyl radicals and cell death (Kohanski et al. 2008). However, for aminoglycosides, such as gentamicin, there is strong evidence for a link between antibiotic uptake provided by the PMF (Taber et al. 1987) and toxicity that is independent of ROS formation. It is suggested that Δψ, the electrical component of the PMF, provides the main driving force for the uptake of positively charged aminoglycosides (Taber et al. 1987; Farha et al. 2013). Indeed, aminoglycoside uptake has been shown to be related to Δψ in *E. coli*, *Staphylococcus aureus* and *Bacillus subtilis* (Taber et al. 1987).

There are a number of well-characterised uncouplers of the bacterial PMF, such as 2,4-dinitrophenol (DNP) and carbonyl cyanide *m*-chloromethoxyphenyl hydrazone (CCCP) (Lewis et al. 1994). DNP acts as a proton ionophore providing a path to transport protons across the membrane uncoupling the PMF (Gage and Neidhardt 1993). Dissipation of the electrical component of the PMF in *E. coli* K-12 using DNP has been shown to inhibit dihydrostreptomycin uptake and susceptibility (Campbell and Kadner 1980). The respiratory inhibitor nitric oxide (NO) provides resistance to gentamicin in pathogenic *E. coli* (Ribeiro et al. 2021) and *Salmonella enterica* (McCollister et al. 2011), and the latter study attributed an observed decrease in gentamicin uptake resulted from NO-mediated inhibition of the terminal oxidase complexes of the respiratory chain (McCollister et al. 2011). While there is considerable evidence in separate studies to implicate both the maintenance of PMF and ROS production by the respiratory chain as potentiators of aminoglycoside lethality, there is a notable lack of comparative studies where the two mechanisms are assessed side by side. Hence, the current work sought to assess how the interplay between ROS, PMF and respiratory activity affects the lethality of gentamicin.

## Materials and methods

### Bacterial isolates

EC958 is a well-characterised *E. coli* O25: H4-ST131 multidrug-resistant urosepsis-causing strain that is globally disseminated and resistant to multiple classes of antibiotics (Nicolas-Chanoine et al. 2008; Croxall et al. 2011; Totsika et al. 2011; Forde et al. 2014).

### Aerobic growth conditions

*E. coli* EC958 ST131 wild type was grown in 10 mL LB in a 50 mL falcon tube at 37 °C and 180 rpm overnight to stationary phase. The stationary phase cultures were used to inoculate (at 1% v/v) M9 minimal medium (16 g/L Na_2_HPO_4_.2H_2_O, 3 g/L KH_2_PO_4_, 0.5 g/L NaCl, 1 g/L NH_4_Cl, 0.24 g/L MgSO_4_, 0.01 g/L CaCl_2_, and 4 g/L glucose) supplemented with 0.1% casamino acids (w/v). Cultures were incubated at 37 °C and 180 rpm until mid-exponential phase before use in the following experiments.

### Catalase assay

*E. coli* EC958 ST131 wild-type cultures were grown using the aerobic growth conditions splitting at mid-exponential phase (approx 1.6 × 10^8^ cells/mL) into 60 mL suspensions in 250 mL conical flasks. These suspensions were exposed to DNP (0 or 1 mM), hydrogen peroxide (0.1 mM) or varying concentrations of gentamicin for 30 min at 37 °C. Cell suspensions were harvested (5000 x*g* for 5 min at 4 °C) and washed in 50 mM Potassium Phosphate buffer a total of 3 times before sonication at 18,000 Hz for 5 × 30 s intervals with 30 s incubation on ice in between. Cell debris was removed via centrifugation at 15,000 rpm for 20 min at 4 °C. For the catalase assay, 50 μL of cell-free supernatant was added to 950 μL of 0.036% (w/w) H_2_O_2_ in a quartz cuvette and the linear decrease in absorbance at 240 nm was measured that corresponded to consumption of H_2_O_2_. Conversion of ΔA240 min^−1^ to (μmoles H_2_O_2_)min^−1^ was achieved using the extinction coefficient *ε*_240_ = 36 M^−1^ cm^−1^ (Anderson et al. 1995). A Markwell assay (Markwell et al. 1978) was used to determine protein concentrations to convert units to μmoles H_2_O_2_ min^−1^(mg protein)^−1^. Three biological repeats were carried out for each condition with three technical repeats completed for each biological repeat.

### Native-PAGE KatE and KatG catalase assay

The same *E. coli* EC958 ST131 wild-type cell extract samples used for the catalase assay exposed to 0 μg/mL gentamicin, 100 μg/mL gentamicin or 0.1 mM H_2_O_2_ were also used for the Native-PAGE (polyacrylamide gel electrophoresis) *KatE* and *KatG* assay. Native-PAGE gels used composed of 5% stacking and 6% resolving acrylamide gels. The exact gel components, loading sample buffer and running buffer used are as described in (Pezzoni et al. 2018). A Markwell assay (Markwell et al. 1978) was used to determine protein concentrations with 10 μg of cell extract loaded into each lane. For the positive control, 10 units (0.26 ng) of bovine catalase were used (one unit of catalase will decompose 1 µmole of H_2_O_2_ per minute at pH 7.0 at 25 °C). A Bio-RAD Mini-PROTEAN Tetra Cell was used to run two identical gels at 15 mA for 2 h 45 min at 4 °C. One gel was stained with Coomassie stain (1 g/L Coomassie Brilliant blue R, 40% methanol, 10% acetic acid and 50% ddH_2_O) overnight before applying de-stain (40% methanol, 10% acetic acid and 50% ddH_2_O) and then imaging use a G-box gel doc system. The second identical gel was stained for catalase activity. This gel was soaked in distilled water for 5 min before incubating with 100 mL 4 mM H_2_O_2_ for 10 min at room temperature. After H_2_O_2_ incubation, the gel was washed with distilled water before soaking in a 100 mL solution containing 1% (w/v) ferric chloride and 1% (w/v) potassium ferricyanide (III) at room temperature. As soon as the gel turned dark green (~ 5 min), the ferric chloride and potassium ferricyanide solutions were removed and the gel rinsed with distilled water before imaging. ImageJ v1.53 was used to analyze the band intensities of the KatE/G bands. The native PAGE gel image was converted to a TIF (8-bit) (greyscale) with each lane seperated into an equal strip and a profile plot generated for each. The areas of the KatE/G peaks were determined from the profile plots and reported as band intensity.

### ROS flow cytometer assay

*E. coli* EC958 ST131 wild-type cultures were grown using the aerobic growth conditions to mid-exponential phase when suspensions of 10^8^ cells/mL were prepared in fresh M9 minimal medium supplemented with 0.1% casamino acids (w/v). These suspensions were incubated at 37 °C for 30 min and then mixed with 1X ROS dye (Thermo Fisher Total Reactive Oxygen Species Assay Kit 520 nm – catalog number = 88-5930-74), a DCFDA analogue-based dye that is well-suited for detection of free radical containing ROS such as superoxide. Aliquots (200 μL) of the ROS-dyed suspension were added to a foil covered Greiner Bio-One 96-well, F-bottom (chimney well) microplate. Microplates were incubated at 37 °C for 30 min before exposing to gentamicin (100 μg/mL), milliQ water (for no treatment negative control sample) or 1 mM H_2_O_2_ (positive control). A 20 μL sample was taken at 30 and 90 min after exposure. Samples were diluted 1:10 in Phosphate-Buffered Saline (PBS) and fixed with DAPI (5 µg/mL) in 4% PFA (paraformaldehyde)–PBS solution. A BD FACSJAZZ™ flow cytometer was used to analyse the fixed samples capturing 100,000 events for each sample. DAPI was excited by a 405 nm laser and detected with a 450/50 nm band-pass emission filter. ROS dye was excited by a 488 nm laser and detected by a 513/17 nm band-pass emission filter. A two-gate system partially adapted from (McBee et al. 2017) was used to analyse the samples (Supplemental Data, Fig. S1) using NovoExpress 1.3.0 software. The first gate used FSC (Forward Scatter) against FSC-Width to determine single cells, removing doublets and debris. All events that passed the first gate were then screened by the second DAPI gate to identify intact cells (DAPI binds to DNA). Fluorescent intensity of the ROS dye was then measured in all cells that passed the DAPI gate (minimum of 60,000) and mean values reported.

### DNP gentamicin susceptibility assays

For survivability assays under aerobic conditions, *E. coli* EC958 ST131 wild-type cultures were grown using the aerobic growth conditions to mid-exponential phase when suspensions of 10^8^ cells/mL were prepared in fresh M9 minimal medium supplemented with 0.1% casamino acids (w/v). These suspensions were exposed to DNP (0 or 1 mM) for 30 min at 37 °C before incubation with various concentrations of gentamicin for 90 min. After gentamicin exposure, serial dilutions were carried out in 1X PBS and cells were spotted on LB agar in triplicate for overnight incubation at 37 °C for colony counting the next day. Six repeats including two biological repeats were performed for each condition.

For survivability assays under anaerobic conditions, *E. coli* EC958 was grown in 10 mL M9 minimal media supplemented with 0.1% casamino acids (w/v), 100 mM sodium nitrate and 0.2% glycerol (v/v) in a 50 mL falcon tube at 37 °C and 180 rpm aerobially overnight to stationary phase. The stationary phase cultures were used to inoculate (1% v/v) 30 mL of M9 minimal media supplemented with 0.1% casamino acids (w/v), 100 mM sodium nitrate and 0.2% glycerol (v/v) in sealed serum bottles. Cultures were then sparged with nitrogen gas for 3 min each before incubation in a static incubator at 37 °C to until mid-exponential phase. Cultures were then diluted to 10^8^ cells/mL in serum bottles and pre-exposed to ± 1 mM DNP for 30 min before incubation with varying concentrations of gentamicin for 90 min at 37 °C in a static incubator. Cultures were re-sparged with nitrogen gas for 1.5 min both after DNP and gentamicin addition to maintain anaerobic conditions. After gentamicin exposure, serial dilutions were carried out in 1X PBS and cells were spotted on LB agar in triplicate for overnight incubation at 37 °C for colony counting the next day. Six repeats including two biological repeats were performed for each condition.

### Assay of the membrane electrical potential

*E. coli* EC958 ST131 wild-type cultures were grown using the aerobic growth conditions to mid-exponential phase when suspensions of 10^8^ cells/mL were prepared in fresh M9 minimal medium supplemented with 0.1% casamino acids (w/v). These suspensions were exposed to DNP (0 or 1 mM) or GSNO (0 or 15 mM) for 30 min at 37 °C. Cells were harvested (4500 xg for 3 min) and washed in 5 mM HEPES buffer pH 7.2 containing 5 mM glucose a total of 3 times. The reaction buffer was added to a Greiner Bio-One 96-well, F-bottom (chimney well), black, fluotrac, medium binding microplate so that the final concentration of the reaction buffer components after addition of cells is 5 mM HEPES buffer, pH 7.2 with 5 mM glucose, 100 mM KCl and 2 μM of DISC3. The fluorescence was monitored on a FLUOstar Omega plate reader for 60 s at 30 s intervals using excitation and emission wavelengths of 584 nm and 655 nm, respectively, to gather a baseline. Cells were then added at a final concentration to 4 × 10^7^ cells/mL in volume 200 μl. Fluorescence was then monitored for a total of 45 min at 30 s intervals. 200 μl of 5 mM HEPES buffer with 5 mM glucose was used for the negative controls.

### Oxygen consumption assay for DNP

*E. coli* EC958 ST131 wild-type cultures were grown using the aerobic growth conditions to mid-exponential phase when cells were harvested (5000 xg for 5 min) and re-suspended in fresh M9 minimal media supplemented with 0.1% casamino acids (w/v). A Rank Brothers oxygen electrode was baselined using 4 mL of distilled water and calibrated with sodium dithionite essentially as previously described (Gilberthorpe and Poole 2008) using the following parameters: polarizing volts = 0.6, stirring to setting 6, 37 °C. Voltage data were then recorded until a reading of 0 V was reached. The concentration of oxygen for the air-saturated growth media was 200 μM as previously used (Gilberthorpe and Poole 2008; Shepherd et al. 2010). 3.6 mL of M9 minimal medium supplemented with 0.1% casamino acids (w/v) was added to the chamber and the baseline voltage was recorded for 5 min before cells were added to a final concentration of 8 × 10^7^ cells/mL. Voltage data were gathered for 8 min before the plunger was inserted to eliminate external oxygen for 10 min prior to addition of 1 mM DNP or ethanol (5% v/v) for the negative control.

### Oxygen Consumption assay for FCCP

An oxygen consumption assay was carried out on *E. coli* EC958 ST131 wild-type cultures using 10 μM carbonyl cyanide-*p*-trifluoromethoxyphenylhydrazone (FCCP). The assay was the same as for DNP apart from a 2 mL final volume being used instead of 4 mL in the electrode due to limited FCCP being available.

### Anaerobic growth conditions

*E. coli* EC958 was grown in 10 mL M9 minimal media supplemented with 0.1% casamino acids (w/v), 0.2% glycerol (v/v) and ± 100 mM sodium nitrate (to provide the exogenous electron acceptor) in a 50 mL falcon tube at 37 °C and 180 rpm aerobially overnight to stationary phase. The stationary phase cultures were used to inoculate (0.2% v/v) 30 mL of M9 minimal media supplemented with 0.1% casamino acids (w/v), 0.2% glycerol (v/v) and ± 100 mM sodium nitrate in sealed serum bottles. Cultures were then sparged with nitrogen gas for 3 min and incubated at 37 °C in a static incubator. Samples (1 mL) from 3 biological repeats were taken every hour for 8 h and then again after 24 h and OD_600_ was measured on a Cary 60 UV–Vis spectrophotometer (Agilent Technologies).

## Results

### Gentamicin elicits a small increase in ROS in aerobically-grown *E. coli*

**Fig. 1:**
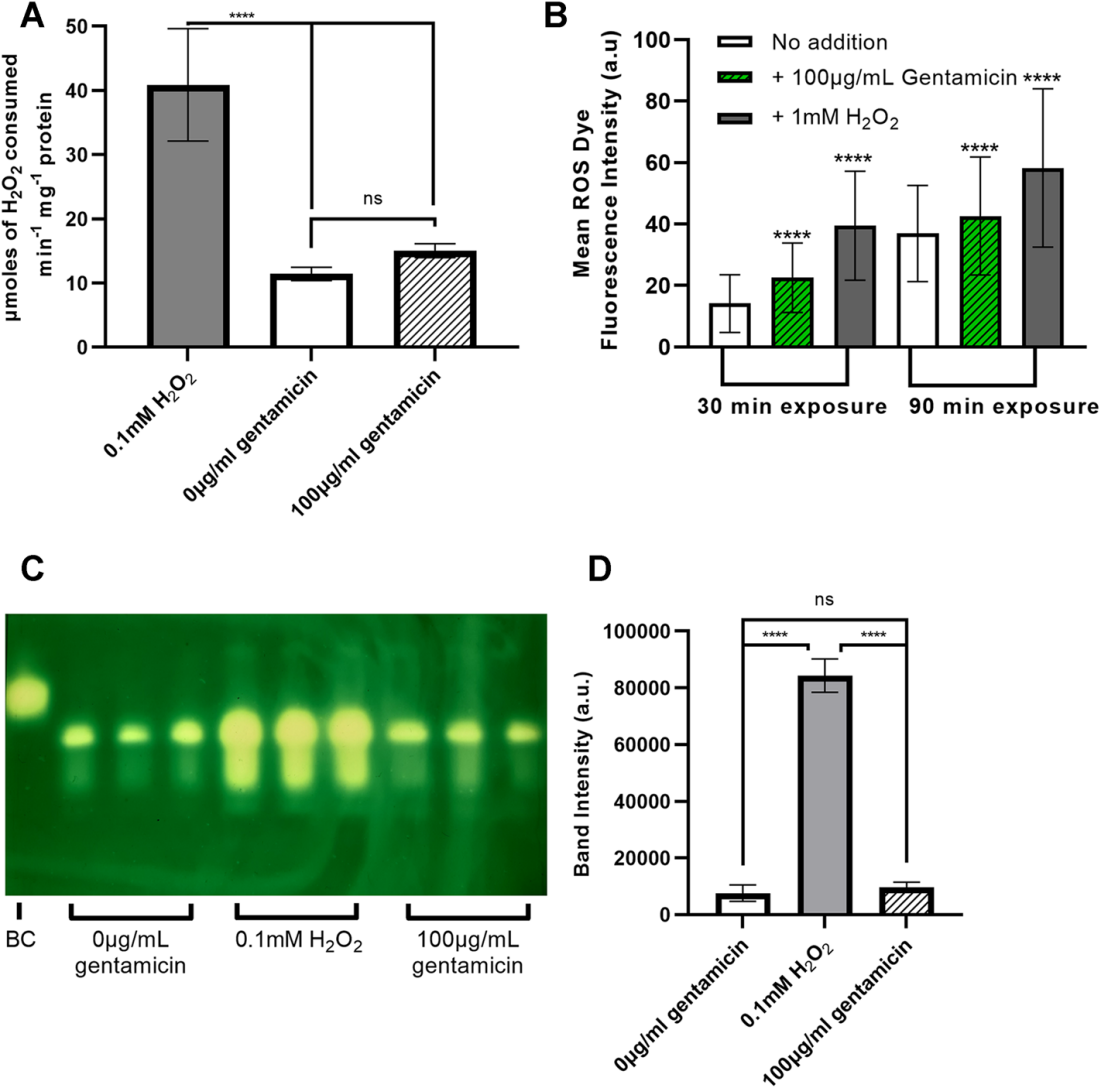
Gentamicin elicits a minimal increase in reactive oxygen species in aerobically grown *E. coli*. **A** Suspensions of 1.6 × 10^8^ cells/ml of *E. coli* EC958 grown in M9 minimal medium supplemented with 0.1% casamino acids were exposed to a sub-lethal level of hydrogen peroxide (0.1 mM), 0 μg/mL gentamicin or 100 μg/ml gentamicin for 30 min with three biological repeats each with three technical repeats completed for each. Cells were then washed, sonicated, cell debris was removed and catalase activity was measured in the cell-free extract (One-way ANOVA with Tukey’s post hoc tests: ****P value < 0.0001, n.s (not significant): *P* value > 0.05). **B** A fluorescent sensitive dye was used to detect ROS in aerobically growing *E. coli* EC958 using a BD FACSJAZZ™ flow cytometer. Whole cells were selected using DAPI staining and gentamicin-mediated changes in fluorescence were analysed using fluorescent intensity of the ROS dye. At least 60,000 cells were sampled for each condition with mean values displayed. (****One-way ANOVA with Tukey’s post hoc tests: *P* value < 0.0001). **C** Native PAGE catalase activity gel. *E. coli* EC958 cells exposed and extract prepared as in **A**. 10 μg of each cell extract was loaded from cells exposed to hydrogen peroxide (0.1 mM), 0 μg/mL gentamicin or 100 μg/ml gentamicin. 10 units of bovine catalase (BC) loaded as a positive control. Gel exposed to 4 mM HO_2_ before staining with ferric chloride–potassium ferricyanide solution. **D** ImageJ analysis of band intensities conducted on the KatE/KatG activity bands (One-way ANOVA with Tukey’s post hoc tests: ****P value < 0.0001, n.s (not significant): *P* value > 0.05)

Previous studies have reported that gentamicin leads to increased ROS levels in nonpathogenic *E. coli* with H_2_O_2_ being the most notable increase, although these statistically significant increases were marginal and drastically lower than for other antibiotics (Dwyer et al. 2014). Hence, it was of interest to quantify gentamicin-mediated increases in ROS in the multidrug-resistant *E. coli* clinical isolate, with a particular focus on H_2_O_2_. The first approach was an indirect measure using a catalase assay to measure the catalase activity in the cytosol (*KatE* and *KatG*) by the decomposition of H_2_O_2_ (Fig. 1A): if elevated peroxide is present then these cells will express higher levels of catalase. Catalase–peroxidase hydroperoxidases I (KatG) and II (KatE) are both known to be located in the cytoplasm (Hillar et al. 1999; Kumar and Imlay 2013). NADH peroxidase (*ahpCF*) can also scavenge H_2_O_2_ and is located in the cytoplasm (Kumar and Imlay 2013). To account for the known effects of gentamicin on protein translation, a markwell assay was first conducted to allow the catalase assay results to be adjusted to the total protein present in the cell extract. The total protein level in the cell extract was lower for the 100 μg/mL gentamicin-exposed sample (58% of the the 0 μg/mL gentamicin-exposed sample). One-way ANOVA results showed there was a significant difference between the three treatment groups of the the catalase assay (*F*(2,24) = 88.34, *P* < 0.0001) (Fig. 1A). As a positive control, exposure to H_2_O_2_ (0.1 mM) was shown to result in a fourfold increase in catalase activity compared to the 0 μg/mL gentamicin sample (Tukey’s post hoc test *P* < 0.0001). However, on addition of 100 μg/mL gentamicin, only a small increase in catalase activity was observed that is not statistically significant (Tukey’s post hoc test *P* = 0.3117). As an alternative approach to measure gentamicin-mediated ROS generation, a 2′,7′-dichlorodihydrofluorescein diacetate-based fluorescent dye was used to directly measure the ROS present in a flow cytometer. A small increase in ROS production can be seen at both 30 min and 90 min after the addition of 100 μg/mL gentamicin (Fig. 1B) compared to the negative control sample (+ milliQ H_2_O). A native PAGE gel catalase assay using the cell extract from (Fig. 1A) was then carried out as a more direct method of measuring the KatE and KatG expression levels (Fig. 1C + D). Cell extract from *E.coli* exposed as in Fig. 1A to 0 μg/mL gentamicin, 0.1 mM H_2_O_2_ or 100 μg/mL gentamicin was run on a native PAGE gel before incubating the gel with H_2_O_2_ and then stained by a ferric chloride–potassioum ferricyanide (III) solution. Potassium ferricyanide (III) reacts with H_2_O_2_ being reduced to potassium ferrricyanide (II). Ferric chloride then reacts with potassium ferrricyanide (II) to form a blue/green insoluble precipitate. This reaction does not happen where KatE and KatG have decomposed the H_2_O_2_ leaving a white band proportional in size to KatE and KatG activity. For a loading control, an additional identical native PAGE gel was run and stained with Coomassie blue which displayed identical loading of each cell extract sample (Supplemental Data, Fig. S6). The bovine catalase-positive control produced a bright band on the catalase activity gel (Fig. 1C) but did not show on the Coomassie-stained gel (Supplemental Data, Fig. S6) likely due to the low amount loaded (0.26 ng) being below the detection limit. *E. coli* KatG is 320 kDa whilst KatE is 336 kDa with the theoretical pI calculated from uniprot being 5.14 for KatG and 5.54 for KatE. As these values are close, KatE and KatG are likely to migrate to a similar point on the native PAGE gel. *E.coli* KatG has previously been shown to migrate further into a native-PAGE gel than KatE (Dong et al. 2009) which would be expected due to the lower molecular weight and lower pI. The brighter band is likely therefore to be KatE whilst the duller band below is likely to be KatG. A clear increase in KatE/KatG activity is seen when exposed to exposure to H_2_O_2_ (0.1 mM). A small increase in catalase activity is also seen in the 100 μg/mL gentamicin-exposed samples compared to the 0 μg/mL gentamicin-exposed samples. However, analysis on the band intensities (Fig. 1D) again showed this to be not statistically significant (One-way ANOVA (*F*(2,6) = 376.8, *P* < 0.0001, Tukey’s post hoc test *P* = 0.7776).

## Dissipation of the proton motive force diminshes gentamicin lethality in aerobially-grown cells

**Fig. 2:**
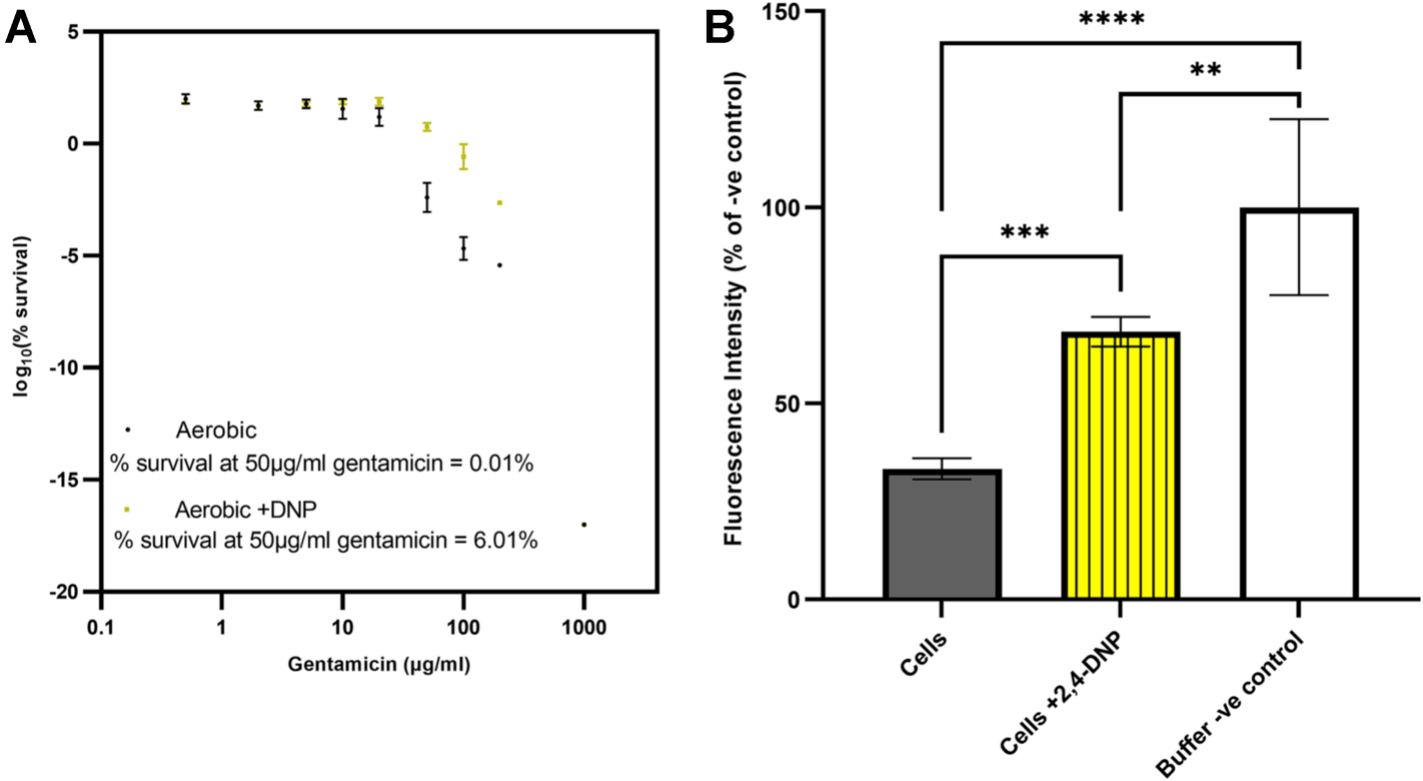
Dissipation of the proton motive force leads to a significant decrease in gentamicin toxicity **A** *E. coli* EC958 cells grown aerobically in M9 minimal media supplemented with 0.1% casamino acids were exposed to ± 1 mM DNP for 30 min followed by incubation with varying concentrations of gentamicin for 90 min. This was followed by serial dilutions, spotting on LB agar and overnight incubation at 37 °C to enumerate CFU/mL. Mean % survival values are shown ± standard deviation from 2 biological repeats each with 3 technical repeats. **B** *E. coli* EC958 cells grown aerobically in M9 minimal media supplemented with 0.1% casamino acids were exposed to ± 1 mM DNP for 30 min. Cells were harvested and the electrical potential of the PMF was measured with a plate reader using the fluorescent dye DiSC_3_(5) (2 µM). Control experiments were performed without DNP and without cells (i.e. buffer only). Data in the figure are at time point 2452 s and are averages of six repeats including two biological repeats for each condition. Data expressed as % relative to the negative control. (One-way ANOVA with Tukey’s post hoc tests: ****P value < 0.0001, ***P value < 0.001, **P value < 0.01)

The effect of the PMF uncoupler DNP on gentamicin susceptibility was tested on aerobically grown *E. coli* EC958 cells. Exponentially growing cells were pre-exposed to 1 mM DNP for 30 min before addition of varying gentamicin concentrations. The presence of DNP resulted in a significant decrease in gentamicin lethality (Fig. 2A). The IC_50_ value without DNP was 5 μg/mL which is consistent with the EUCAST MIC values for gentamicin and *E. coli* at > 4 mg/L (Jakobsen et al. 2007). The IC_50_ value with DNP was 45 μg/mL resulting in a 600-fold (3.15 log10) increase in % survival at 50 μg/mL gentamicin.

To confirm that DNP is indeed dissipating the PMF, a fluorescent voltage-sensitive dye DiSC_3_(5) was used to measure the electrical component of the PMF: DiSC_3_(5) associates with the negatively charged inner membrane when the PMF is high, and is released upon membrane depolarization resulting in an increase in fluorescence (te Winkel et al. 2016). *E. coli* EC958 ST131 was again pre-exposed to 1 mM DNP for 30 min before PMF quantitation with DiSC_3_(5), and a positive control was performed for ‘cells without DNP’ (Fig. 2B). A negative control was performed without cells or DNP (i.e. buffer only) to measure the maximum fluorescence of the free dye. A clear increase in fluorescence is observed of approximately twofold between cells exposed to DNP and those not exposed to DNP. Since the NO-donor GSNO, a respiratory inhibitor, has previously been shown to provide resistance to gentamicin in the pathogenic *E. coli* EC958 ST131 strain (Ribeiro et al. 2021), it was also of interest to confirm that GSNO can dissipate the PMF, which indeed it can (Supplemental data, Fig. S2).

## The uncoupler DNP does not alter respiratory oxygen consumption but results in an increase in ROS production

**Fig. 3:**
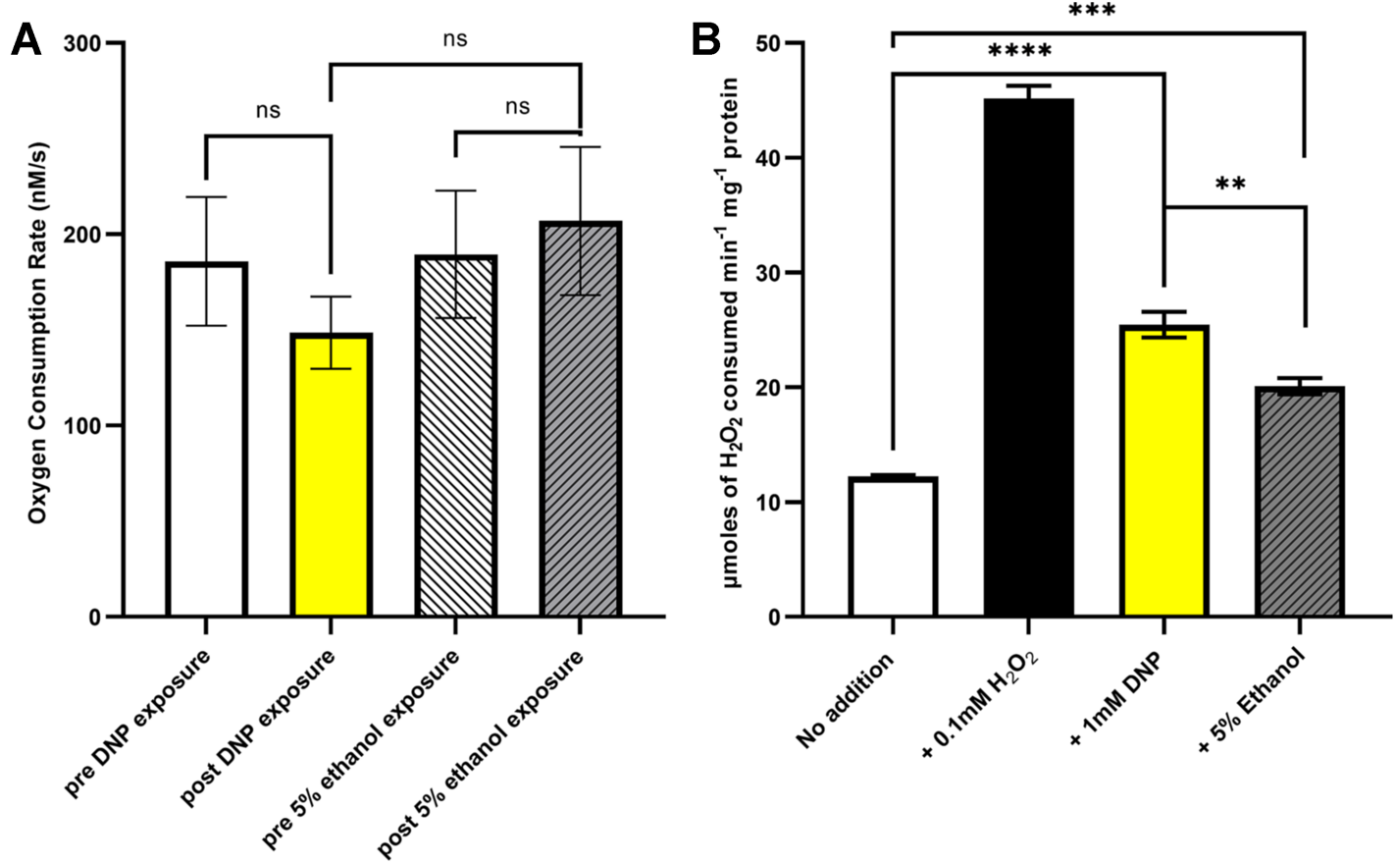
Dissipation of the proton motive force does not alter respiratory oxygen consumption but increases ROS. **A** Suspensions of 4.8 × 10^8^ CFU/mL of *E. coli* EC958 grown aerobically were diluted into fresh M9 minimal medium supplemented with 0.1% casamino acids to 8.0 × 10^7^ CFU/mL in the Rank Brothers Clark oxygen electrode chamber after a baseline of 5 min was recorded. Voltage data were collected for 8 min followed by the addition of the plunger to exclude external oxygen and data were recorded for another 10 min. DNP (1 mM final) was injected and data recorded until all oxygen was consumed. Since, DNP was dissolved in ethanol, control experiments were performed with this solvent. (One-way ANOVA with Tukey’s post hoc tests: n.s (not significant): *P* value > 0.05). **B** Suspensions of 1.6 × 10^8^ cells/ml of *E. coli* EC958 grown in M9 minimal medium supplemented with 0.1% casamino acids were exposed to a sub-lethal level of hydrogen peroxide (0.1 mM), ± 1 mM DNP or 5% ethanol for 30 min. Cells were then washed, cell debris was removed and catalase activity was measured in the cell-free extract. (One-way ANOVA with Tukey’s post hoc tests: ****P value < 0.0001, ***P value < 0.001, **P value < 0.01)

The influence of 1 mM DNP upon the respiration rate of *E. coli* EC958 was measured via monitoring oxygen consumption in an oxygen electrode (Fig. 3A, Supplemental Data Fig. S3). A minor decrease in oxygen consumption of aerobically grown cells was observed in response to 1 mM DNP that is not statistically significant (One-way ANOVA (*F*(3, 8) = 1.76, *P* = 0.2322). An ethanol control was also performed as DNP was solubilised in this solvent, which elicited a small rise in oxygen consumption that was not statistically significant. (One-way ANOVA (*F*(3, 8) = 1.76, *P* = 0.2322)).

The influence of the alternative more potent uncoupler FCCP upon the respiration rate of *E. coli* EC958 was also studied (Supplementary data Fig. S5). At 10 μM, only a minor increase in the respiration rate was seen that is not statistically significant (unpaired t test *P* = 0.7871).

Interestingly, DNP treatment produced a noticeable increase in catalase activity (Fig. 3B) indicating that DNP is promoting the production of ROS species (One-way ANOVA (*F*(3,7) = 734.9, *P* < 0.0001, Tukey’s post hoc test *P* < 0.0001). The ethanol control also resulted in an increase in catalase activity, although DNP exposure did result in a statistically significant increase in catalase activity compared to this control [One-way ANOVA (*F*(3,7) = 734.9, *P* < 0.0001, Tukey’s post hoc test *P* = 0.0013)].

## Dissipation of the proton motive force diminishes gentamicin lethality in anaerobically grown cells

**Fig. 4:**
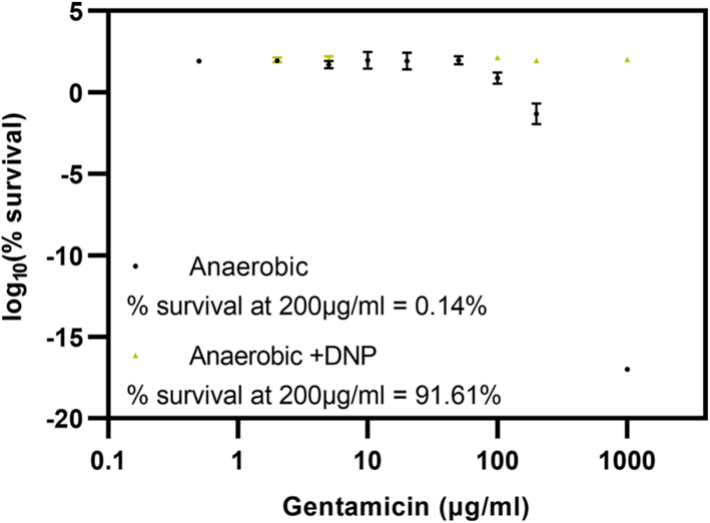
The uncoupler DNP elicits a decrease in gentamicin toxicity under anaerobic conditions. *E. coli* EC958 cells grown anaerobically in M9 minimal media supplemented with 0.1% casamino acids (w/v), 100 mM sodium nitrate and 0.2% glycerol (v/v) were exposed to ± 1 mM DNP for 30 min followed by incubation with varying concentrations of gentamicin for 90 min. This was followed by serial dilutions, spotting on LB-agar and overnight incubation at 37 °C to enumerate CFU/mL. Mean % survival values are shown ± standard deviation from 2 biological repeats each with 3 technical repeats

As demonstrated above, dissipation of the PMF by DNP under aerobic conditions diminishes gentamicin lethality (Fig. 2A), and this effect was not due to alleviation of ROS production by DNP (Fig. 3B): indeed, quite the opposite. To analyse the effects of DNP-mediated PMF disruption upon gentamicin lethality in isolation from ROS, viability assays were performed under anaerobic conditions (i.e. where ROS production by the respiratory chain is excluded). To ensure that cells were performing anaerobic respiration, control experiments were performed in the absence of exogenous electron acceptors which abolished growth (Supplemental Data, Fig. S4). Pre-exposure of *E. coli* to 1 mM DNP for 30 min at OD_600_ = 0.1 prior to addition of varying gentamicin concentrations resulted in a large decrease in gentamicin lethality (Fig. 4). The change in IC_50_ value resulting from DNP exposure could not be quantified due to the complete abolition of gentamicin toxicity by DNP, although it was noted that in the absence of DNP gentamicin toxicity was diminished (IC_50_ = 91.19 µg/mL) compared to aerobic growth (IC_50_ = 5 μg/mL, Fig. 2A). This might suggest a role for anaerobic respiratory complexes in gentamicin lethality, although this is speculative at this stage and will require further work to investigate this hypothesis.

## Discussion

It is well-known that respiratory activity can promote the lethality of many antibiotics, and there is unequivocal evidence to support the role of ROS generation by the aerobic respiratory chain in the lethality of a number of different antibiotic classes (reviewed in (Stokes et al. 2019)). However, the interplay between ROS, respiratory activity and aminoglycoside lethality has remained less clear, and the maintenance of PMF provides an alternative mechanism for the respiratory chain to potentiate the lethality of aminoglycosides (i.e. via promoting the uptake of positively charged aminoglycosides). Hence, the current study sought to investigate the competing ROS *vs.* PMF hypotheses under comparable conditions to assess the underpinning mechanism for gentamicin lethality for a clinically-relevant *E. coli* strain. The first question to answer was: does exposure of *E. coli* to gentamicin result in the production of ROS? To answer this simple question, the three approaches of a catalase assay, direct ROS measurement and a native PAGE KatE/KatG catalase assay were undertaken and all approaches demonstrated that gentamicin exposure does not result in a significant increase in ROS under the conditions tested (Fig. 1). It has previously been shown that the 30 min exposure to 0.1 mM H_2_O_2_ used in this investigation can upregulate *E. coli* KatG and increase survivability against oxidative stress (Rodríguez-Rojas et al. 2020). The experiments were performed over a concentration range that is known to be toxic to this strain under the conditions tested and provides very good evidence to support the hypothesis that lethal doses of gentamicin do not elicit significant increases in ROS. The small increase in ROS production seen in the direct ROS measurements (Fig. 1B) could be due to the detection of superoxide that the 2′,7′-dichlorodihydrofluorescein diacetate-based fluorescent dye can detect (Freemerman et al. 2014) as opposed to the catalase assays being specific for H_2_O_2_ detection.

The respiratory uncoupler DNP has been previously used to demonstrate that dissipation of the PMF significantly diminishes the lethality of the aminoglycoside dihydrostreptomycin (Campbell and Kadner 1980). To verify that this is the case under the same conditions that ROS species were measured, aerobically grown *E. coli* EC958 was exposed to 1 mM DNP over a range of gentamicin concentrations and viable counts were measured. These data confirm that DNP elicits a significant decrease in gentamicin lethality (Fig. 2A), consistent with the hypothesis that maintenance of the PMF by respiratory activity potentiates the bactericidal effects of gentamicin. To further investigate this, 1 mM DNP was confirmed to dissipate the electrical potential of the inner membrane as expected (Fig. 2B). Since the respiratory inhibitor and NO-donor GSNO has previously been shown to diminish gentamicin lethality (Ribeiro et al. 2021), it was of interest to investigate the hypothesis that exposure to GSNO could dissipate the PMF. This was tested under the conditions used in this previous study, and a significant dissipation of the membrane electrical potential was observed in response to GSNO (Supplemental Data, Fig. S2), a magnitude change comparable to that of 1 mM DNP. Significant effort was made to investigate whether GSNO elevated ROS production using the fluorescent dye approach described herein, but the background fluorescence generated by GSNO was far too high to generate reliable and reproducible results. Furthermore, the catalase assay could not be used as this enzyme utilises a haem cofactor and is well-known to be inhibited by NO (Titov and Osipov 2017). Nevertheless, transcriptomics studies have been undertaken where *E. coli* was exposed to GSNO and no perturbations in ROS stress responses were detected (Flatley et al. 2005). Taken together with the data described herein, this supports the hypothesis that inhibition of aerobic respiration by GSNO diminishes the lethality of gentamicin through dissipation of the PMF and not alleviation of ROS generation by the respiratory chain. Indeed, our observations are consistent with previous work on *Salmonella* where NO-mediated respiratory inhibition diminished the toxicity of aminoglycosides through a mechanism involving diminished gentamicin uptake (McCollister et al. 2011).

It has previously been reported that protonophores, such as FCCP and DNP, can block translocation of periplasmic and membrane proteins in *E. coli* leading to aggregation and induced heat shock response with an increased level of the heat shock regulator protein sigma-32 (Jana et al. 2009). The heat shock protease ClpP is implicated in this process (Jana et al. 2009) and upregulation of the heat shock proteases has been linked to increased resistance to antimicrobial stress in *Acinetobacter baumannii* (Lazaretti et al. 2020)(Cardoso et al. 2010) and *E.coli* (Yamaguchi et al. 2003)((Kohanski et al. 2008). DNP has previously been shown to upregulate several heat shock responses in *E. coli* (Gage and Neidhardt 1993), so it is important to consider that DNP-induced stress response may contribute to the DNP-mediated increases in resistance to gentamicin observed herein (Figs. 2A and 4). However, the well-characterised uncoupling action (and resulting diminished aminoglycoside uptake) seems a more probably explanation for the DNP-mediated decrease in gentamicin lethality.

To further investigate potential influences in oxygen utilisation by DNP, oxygen electrode experiments were performed. These data demonstrate that DNP did not elicit any significant change in respiratory oxygen consumption at 1 mM (Fig. 3A), which is consistent with previous studies using 1 mM DNP (Goto and Anraku 1974) as DNP is a protonophore that allows protons to leak across the normally proton-impermeable cytoplasmic membrane inhibiting PMF-dependent transport systems such as gentamicin uptake with little effect on the cellular respiration (Taber et al. 1987). As a control, the more potent uncoupler FCCP also did not show any significant change in oxygen consumption at 10 μM (Supplemental Data, Fig. S5). We acknowledge that for these experiments cells were exposed to uncouplers for slightly less time than the 30 min pre-exposures used for some other figures, although we do not anticipate that a marginal increase in exposure time would elicit an increase in oxygen consumption. It was interesting to observe an increase in catalase activity in cells that were exposed to DNP, especially as DNP protects the cells against gentamicin lethality (Fig. 2A). Two explanations therefore present themselves: i) The effects of DNP-mediated increase in ROS upon cell survival are insignificant compared to the DNP-mediated decrease in PMF that protect *E. coli* from gentamicin uptake ROS are not; ii) pre-exposure to DNP activates the ROS stress-response systems and renders the cells more tolerant to gentamicin-mediated ROS toxicity possibly through upregulation of KatE and KatG. However, given that gentamicin does not elicit the formation of significant levels of ROS in this study (Fig. 1), it is more likely that the protective effects of DNP-mediated membrane depolarisation (Fig. 2B) outweigh any toxicity issues resulting from elevated ROS due to DNP.

Finally, as aminoglycoside antibiotics work on *E. coli* grown under anaerobic conditions, it is logical to hypothesise that DNP will also diminish the lethality of gentamicin under these conditions. To test this hypothesis, EC958 was grown under anaerobic conditions and viability assays were performed following pre-exposure to 1 mM DNP followed by addition of various concentrations of gentamicin (Fig. 4). These data support the hypothesis that dissipation of the PMF by DNP can abolish the lethality of gentamicin under conditions that exclude the possibility of ROS generation by the respiratory chain. This further supports the hypothesis that DNP diminishes the lethality of gentamicin through dissipation of the PMF and not alleviation of ROS generation by the respiratory chain.

## Supplementary Information

Below is the link to the electronic supplementary material.

Supplementary file1 (DOCX 700 kb)
