# Supplementary material for: Proton motive force underpins respiration-mediated potentiation of aminoglycoside lethality in pathogenic Escherichia coli
Source: Arch Microbiol. 2022 Jan 6;204(1):120. doi: 10.1007/s00203-021-02710-y (PMC8739286; doi:10.1007/s00203-021-02710-y)

**Supplemental Data**

**Figure S1.** **Overview of ROS flow cytometry gating method showing example data.** *(A)* Stage 1 (Forward scatter (FSC) against FSC width) selects for bacterium-sized particles removing doublets, multiples and large debris (Size Gate). Events in the size gate are passed to *(B)* Stage 2 (DAPI gate) which selects for intact cells that contain DNA*. (C)* Stage 3 measures the ROS using the fluorescent intensity of the ROS dye. Data in *(C)* is shown for the 90 min exposure sample to no treatment (+ milliQ water), + 100 μg/mL gentamicin or positive control (+ 1 mM H_2_O_2_). Method was partially adapted from (McBee et al. 2017) (Stages 1 + 2).


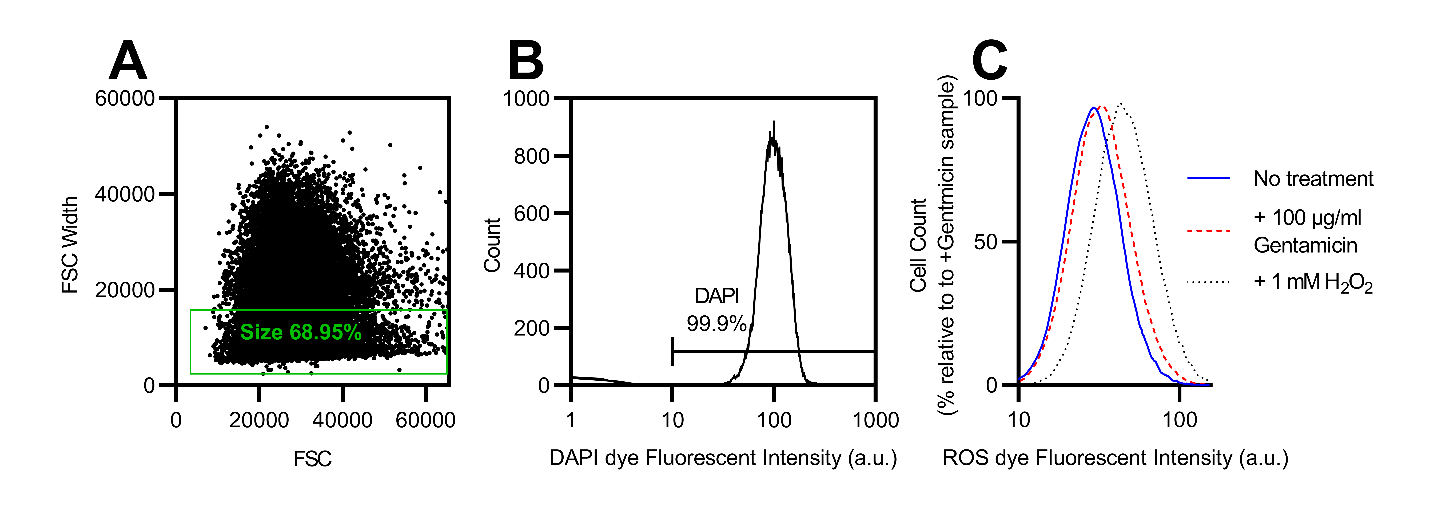


**Figure S2. GSNO dissipates the proton motive force.** Suspensions of 10^8^ cells/mL of *E. coli* EC958 ST131 grown in M9 minimal media supplemented with 0.1 % casamino acids were exposed to +/- 15 mM GSNO for 30 min. Cells were re-suspended in 5 mM HEPES buffer, pH 7.2, containing 5 mM glucose to 1.6 x 10^8^ CFU/mL. 5mM HEPES buffer, pH 7.2, containing 5mM glucose, 100mM KCl, 1% DMSO and 2μM DiSC_3_(5) was added to wells of a 96-well plate and baseline fluorescence was monitored for 90 s in a plate reader using excitation and emission wavelengths of 584 nm and 655 nm, respectively. Cells were then added to a final density of 4 x 10^7^ CFU/mL and fluorescence was recorded every 30 s for a further 2610 s. Data are averages of six repeats including two biological repeats for each condition. Negative control was included of 5 mM HEPES buffer, pH 7.2, containing 5 mM glucose, 100mM KCl, 1% DMSO and 2μM DiSC3(5). Data taken at time point 2452s. Data expressed as % relative to the negative control.

(One-way ANOVA with Tukey post-hoc tests: ***: *P*-value <0.001).


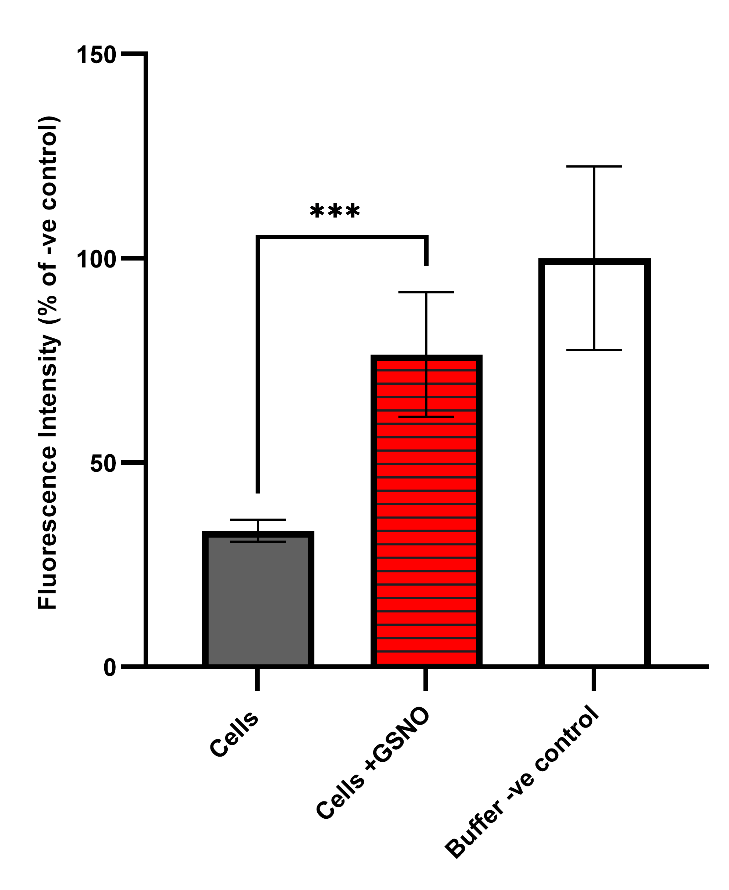


**Figure S3. Measurement of respiratory oxygen consumption.** Suspensions of 4.8 x 10^8^ CFU/mL of *E. coli* EC958 were diluted into fresh M9 minimal medium supplemented with 0.1 % casamino acids to 8.0 x 10^7^ CFU/mL in a Rank Brothers oxygen electrode chamber after a baseline of 5 min was recorded (1). Voltage data was collected for 8 min followed by the addition of the plunger to exclude external oxygen (2) and data were recorded for another 10 min. Injection of 1 mM DNP (dissolved in ethanol) was performed to 5 % of the total volume (3). Conversion from voltage to nM oxygen and nM/s used ΔV = 200 μM O_2_.


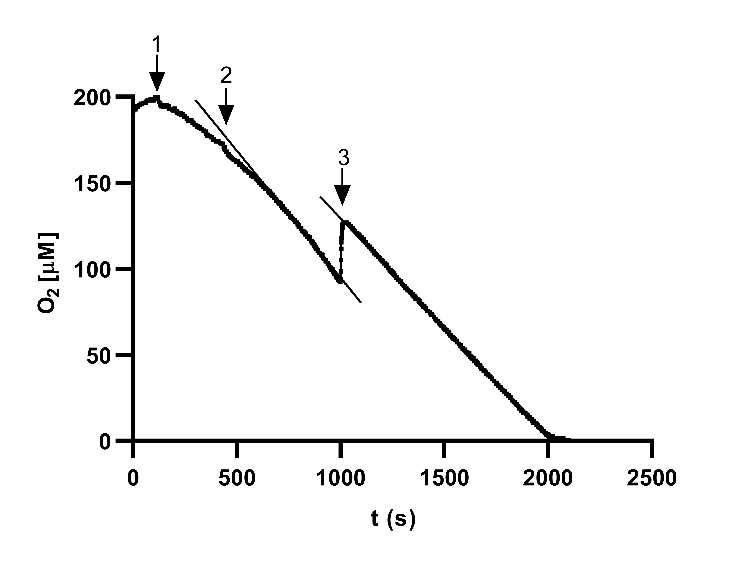


**Figure S4. Lack of exogenous electron acceptor prevents growth in anaerobic conditions.** *E. coli* EC958 was grown under anaerobic conditions in M9 minimal medium supplemented with 0.1 % (w/v) casamino acids (no external electron acceptor) or 0.1 % casamino acids and sodium nitrate (100 mM) to provide the exogenous electron acceptor. Mean OD_600_ values are shown +/- standard deviation from 3 biological repeats.


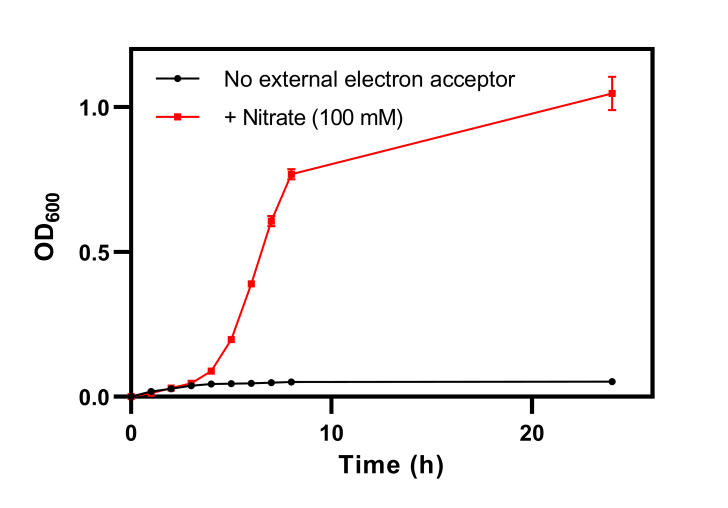


**Figure S5. Measurement of respiratory oxygen consumption in response to FCCP.** Suspensions of 4.8 x 10^8^ CFU/mL of *E. coli* EC958 grown aerobically were diluted into fresh M9 minimal medium supplemented with 0.1 % casamino acids to 8.0 x 10^7^ CFU/mL in the Rank Brothers Clark oxygen electrode chamber after a baseline of 5 min was recorded. Voltage data was collected for 8 min followed by the addition of the plunger to exclude external oxygen and data was recorded for another 10 min. FCCP (10 μM final) was injected and data recorded until all oxygen was consumed. Since, FCCP was dissolved in ethanol, control experiments were performed with this solvent at 5%. (Student’s two-tailed unpaired *t*–test n.s (not significant): *P*-value >0.05)


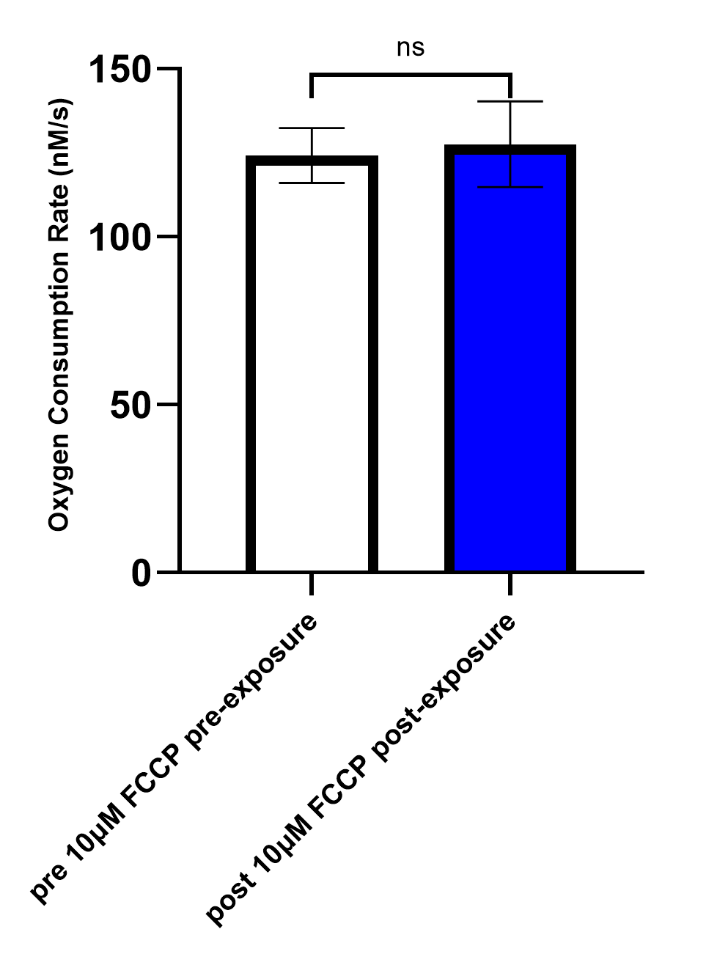


**Figure S6. Coomassie stained Native-PAGE gel.** *E. coli* EC958 cells exposed and extract prepared as in Figure 1A. 10 μg of each cell extract was loaded from cells exposed to hydrogen peroxide (0.1 mM), 0 μg/mL gentamicin or 100 μg/ml gentamicin. 10 units of bovine catalase (BC) loaded as a positive control.


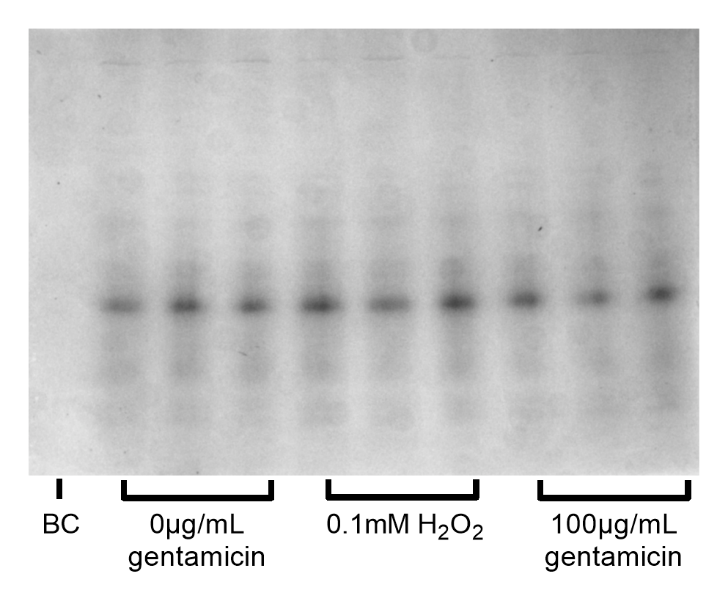

Supplement: Supplementary file 1 — Supplementary file1 (DOCX 700 kb) [file 203_2021_2710_MOESM1_ESM.docx]
